# COVID-19 Predictive Models Based on Grammatical Evolution

**DOI:** 10.1007/s42979-022-01632-w

**Published:** 2023-02-02

**Authors:** Ioannis G. Tsoulos, Chrysostomos Stylios, Vlasis Charalampous

**Affiliations:** 1 Department of Informatics and Telecommunications, University of Ioannina, Arta, Greece; 2 Industrial Systems Institute, Athena Research Center, Patras Science Park Building, Platani, Patras, Greece

**Keywords:** COVID-19, Predictive models, Feature construction, Machine learning, Grammatical evolution

## Abstract

A feature construction method that incorporates a grammatical guided procedure is presented here to predict the monthly mortality rate of the COVID-19 pandemic. Three distinct use cases were obtained from publicly available data and three corresponding datasets were created for that purpose. The proposed method is based on constructing artificial features from the original ones. After the artificial features are generated, the original data set is modified based on these features and a machine learning model, such as an artificial neural network, is applied to the modified data. From the comparative experiments done, it was clear that feature construction has an advantage over other machine learning methods for predicting pandemic elements.

## Introduction

In December 2019, when the first reports emerged of a mysterious infection in the Chinese province, Wuhan, the world entered a new pandemic era. The WHO (World Health Organization) officially declared it a global pandemic on March 11, 2020. The name given to the new coronavirus was SARS-CoV-2 (Severe Acute Respiratory Syndrome) and the name given to the corresponding disease was COVID-19. As Andersen mentioned [1], the virus’s origin is the natural selection in an animal host before the zoonotic transfer or natural selection in humans following the zoonotic transfer. At the time of writing, the number of confirmed cases is over 100,000,000 and the number of confirmed cases has exceeded 2,500,000, while the fatality rate of the confirmed cases is almost 3%. However, the overall fatality rate is estimated to be lower, due to the shortage of testing in some countries and also, because of the asymptomatic patients, that are unlikely to seek a test.

In the current work three distinct experiments on COVID-19 were conducted: in the first experiment, the countries were categorized into seven different classes according to the mortality rate in each country, in the second experiment, the mortality rate in some selected countries is predicted and finally a dataset derived from the Israeli Ministry of Health was used to predict the COVID-19 diagnosis. We used seven classes based on the available data so that every class to have representative number of data. In both experiments an approach for feature construction was utilized. This approach was initially described in [2]. That approach uses the so-called grammatical evolution technique [3] to select and construct a subset of the original features set that satisfies a priori defined classification accuracy. In contrast to many traditional features selection approaches where experts or semi-automatic methods derive or transform the original set of features, the proposed method is fully automatic. The approach for feature selection and creation was used with success on a series of problems such as Spam Identification [4], Fetal heart classification [5], epileptic oscillations in clinical intracranial electroencephalograms [6], etc.

The rest of this article is organized as follows: in “ Related Work” the related work on the topic is presented and discussed, in “Method Description” the proposed method is described in detail as well as the datasets used, in “ Experiments” the experiments of the proposed method are presented and compared with the results from some well-known methods and finally in “Discussion” the proposed method is discussed.

## Related Work

From the onset of the pandemic, scientists from different scientific fields have been focusing their efforts on the puzzle that is COVID-19. More particularly, in Computer Science, many influential papers have been published, where complex mathematical models, artificial intelligence algorithms, and other similar models have been developed, that try to predict the virus’ behavior.

Wang presented the Patient Information Based Algorithm (PIBA), which is based on real-time data collected from patients during the outbreak in Wuhan. With this algorithm [7], he built a model that estimates and predicts death rates in the near future. The fatality rate is not calculated by simply dividing the number of patients by the deaths. The number of deaths that day is divided by the number of potential patients on a day or days when the patient has just started to develop the disease. They used two parameters from the data: the period from the onset of symptoms to death and the period from entry to the intensive care unit (ICU) to death. This PIBA model predicted a fatality rate of 1.6%. In their study, Tomar and Gupta [8] used the data-driven Long Short-Term Memory (LSTM) method and the curve-fitting technique to predict the number of cases in the succeeding days. They used data from January 30 till April 4, 2020, and they predicted the total number of confirmed cases, the total number of recovered patients, the number of daily positive tests, and the number of daily deaths. They concluded that with the above techniques, predictions for beyond 30 days could be performed successfully. Zhang et al. [9], presented a segmented Poisson model to analyze the daily new COVID-19 cases in Canada, France, Germany, Italy, the UK and the USA. They took into consideration the governments’ interventions and they managed to make predictions about the turning point of the pandemic (the time that the daily cases reach a peak), the duration (outbreak period), and the attack rate (percent of the population that will be infected during the outbreak). Al-qannes et al. [10] presented a model for forecasting and estimating confirmed cases in a ten-days time-frame. The collected data was concerned China, for the period 21 January–18 February 2020 and was retrieved from the WHO. An adaptive neuro-fuzzy inference system (ANFIS) was used, with modified enhanced flower pollination (FPA) by using the Salp Swarm Algorithm (SSA). The FPASSA-ANFIS model, they developed, showed good performance and produced good results. Kucharski et al. [11] developed a stochastic transmission model to predict the potential outbreaks of the virus in other areas. They estimated how the transmission might vary over time, and from this, they predicted the potential outbreaks, in other areas. They used data from Wuhan, and more specifically, the datasets they used were: new cases in Wuhan with no market exposure (onset date January 26, 2020), daily new exported cases from the Chinese province (onset date January 26, 2020), daily new cases in China, and the proportion of infected passengers on evacuation flights. They also used some extra datasets for comparison with the model outputs. Pandey et al. [12] presented a prediction model for India, using the SEIR (Susceptible-Exposed-Infected-Recovered) and Regression model. They used data from Johns Hopkins in the period of January 30, 2020 to March 30, 2020 and predicted the number of cases per million for the upcoming two weeks and by using the SEIR model computed the R0 value. Pinter et al. [13] used an ANFIS system and a multi-layered perceptron-imperialist competitive algorithm (MLP-ICA) to predict the mortality rate for Hungary. Smith et al. [14], applied a series of machine learning techniques to a dataset of blood samples taken from COVID-19 patients from a hospital in the region of Wuhan, China. Their goal was to identify the primary factors in predicting the mortality rate and the factors discovered were age, days in the hospital, Lymphocyte and Neutrophils. Zohair et al. [15] studied various machine learning models to estimate the impact of weather variables, such as temperature and humidity, on the transmission of the COVID-19 disease among the population. The experimental results showed that the weather variables could predict the mortality rate. Also, the result indicated that the number of infections decreases as the temperature increases.

## Method Description

### Datasets

During the pandemic, many APIs (Application Programming Interface) with COVID-19 data have made their appearance. Notably, Postman [16] launched an API resource center where anyone can obtain data from all over the world. We used in our study the disease.sh API center [17], which provides data for all the countries of the world. Mainly, it provides, among others, accumulative, daily, per million data for confirmed cases, active cases, critical cases, recovered people, deaths and tests. In our current work we have used data collected from 210 countries and two distinguished datasets were constructed: the first dataset is used for classification and the second one for regression using in both cases the proposed feature construction technique. The first dataset was used to categorize a country into a mortality class using different observations.The second dataset was used to predict the number of deaths in a series of countries with data fitting the number of deaths.

#### Seven Categories Dataset

For each country a pattern containing six features is constructed.

The features have the following meaning:

1. *Continent*: Here the names of each continent was digitized according to the following scheme:
  The value 0 is used for countries in Europe.
  The value 1 is used for countries in North America.
  The value 2 is used for countries in South America.
  The value 3 is used for countries in Asia.
  The value 4 is used for countries in Africa.
  The value 5 is used for countries in Oceania.
2. *Cases per one million*: Here the total number of cases per one million population is used.
3. *Tests per one million*: The performed COVID-19 tests per one million of population.
4. *Active cases per one million*: The active cases per one million of population.
5. *Recovered people per one million*: The metric of people who have recovered from the disease per one million of the population is used.
6. *Critical cases per one million*: The amount of people in critical condition per one million of the population is used here.

**Fig. 1:**
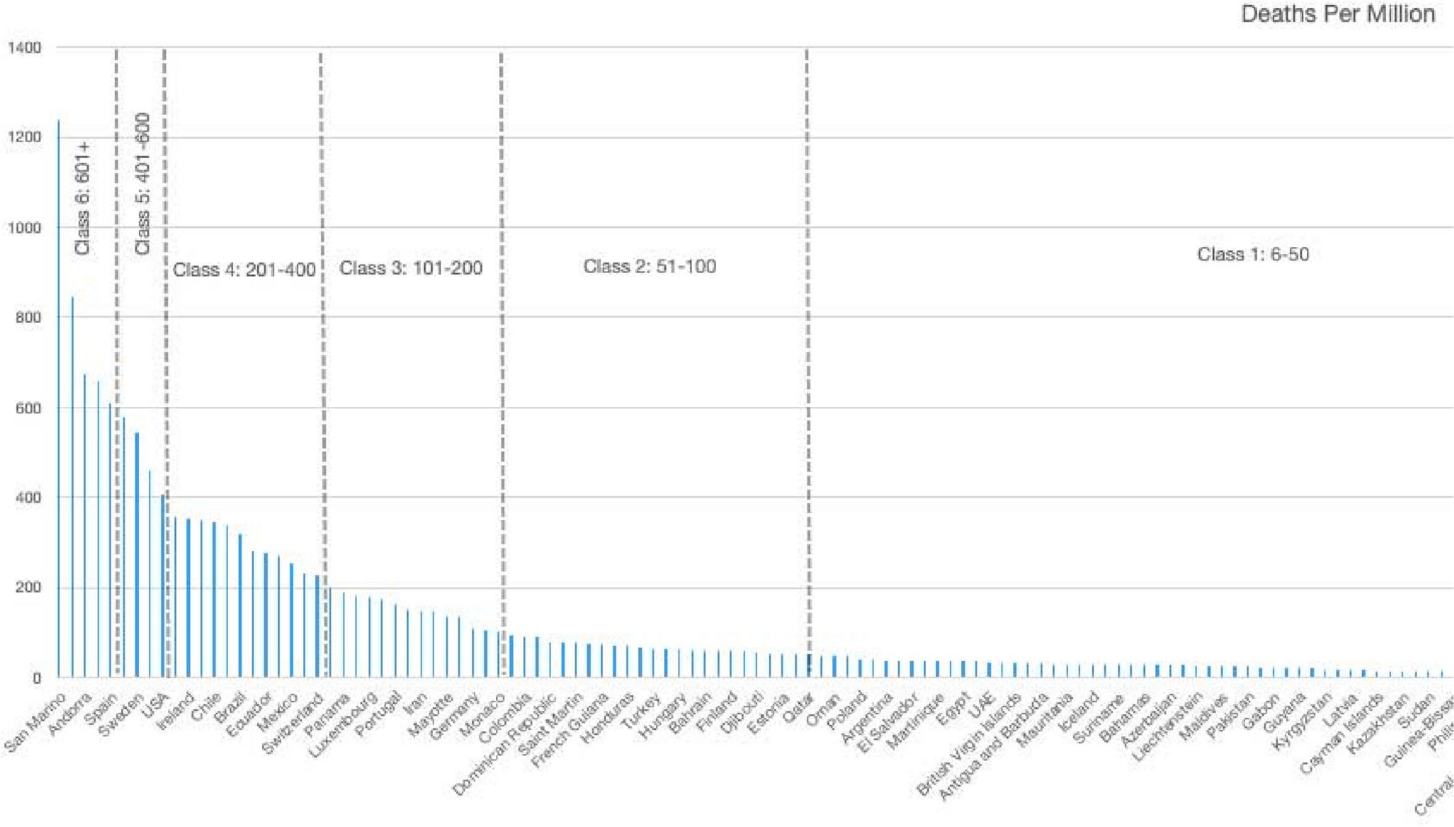
Property profile of the diverse library compared to the compound pool. The horizontal axis represents the state name and the vertical axis is the fraction of deaths per million population

**Fig. 2:**
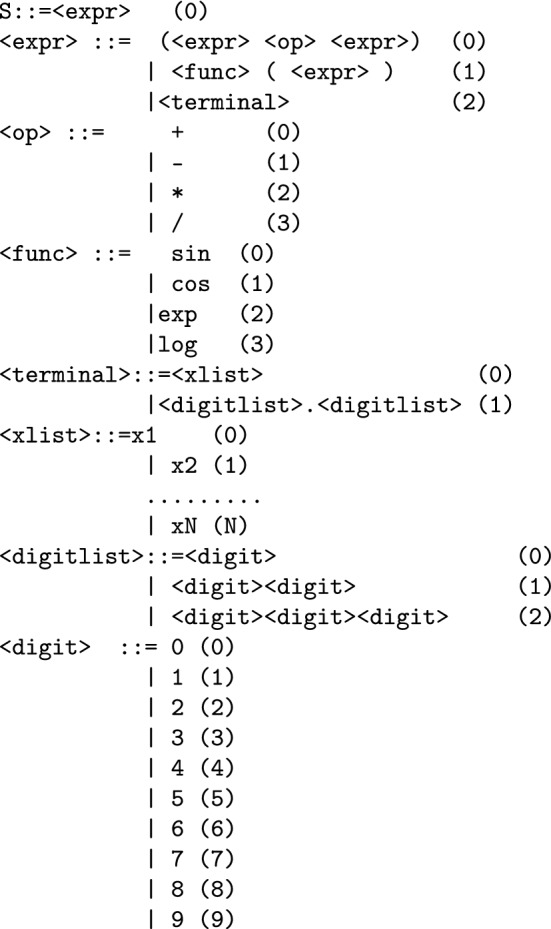
BNF grammar of the proposed method.The numbers in parentheses indicate the serial number of the production rule. Non-terminal symbols are enclosed in $<>$

COVID 19 is a space and time based infection so one of the most important characteristics is the location of the infected population, which is reflecting on the first feature: continent. Then the rate of mortality is dependent on the number of critical cases and recovering cases that are included as feature 5 and feature 6. On the other hand, cases per million (feature 2), tests per million (feature 3) and active cases per million (feature 4) are strongly correlated with feature 5 and feature 6. By using the above information, the goal was to identify the fatality rate for each country. For this purpose, the deaths per one million statistics were used as the class to predict. In order to create classes with a sufficient number of countries in each class the graph in Fig. 1 was constructed and the following partition was made:

1. *Class 0*: Countries that have 0–5 deaths per million. Note that countries with zero fraction are not displayed in the figure.
2. *Class 1*: Countries that have 6–50 deaths per million.
3. *Class 2*: Countries that have 51–100 deaths per million.
4. *Class 3*: Countries that have 101–200 deaths per million.
5. *Class 4*: Countries that have 201–400 deaths per million.
6. *Class 5*: Countries that have 401–600 deaths per million.
7. *Class 6*: Countries that have more than 601 deaths per million.

#### Mortality Prediction Dataset

The second dataset used the number of deaths for the first 180 days of the pandemic for a series of countries. We selected and focused on four countries to test the proposed method. The countries used in this dataset were the following: Belgium, Brazil, France, Germany. The purpose of this experiment is to predict the number of deaths for these countries using machine learning techniques and more specifically to see if using the feature construction technique can make a better prediction than other techniques.

### Feature Construction with Grammatical Evolution

Grammatical evolution is a genetic algorithm where the chromosomes represent production rules of a Backus Naur Form (BNF) grammar as a vector of integer values. The production procedure begins from the start symbol of the grammar and gradually produces the program string, by replacing non terminal symbols with the right hand of the selected production rule. The selection is performed in two steps: initially, the first element from the vector is taken (denoted as V). Subsequently, the production rule is selected using the scheme Rule = *V* mod *R*, where *R* is the number of production rules for the current non terminal symbol.

In the current work the BNF grammar of Fig. 2 is used to create a new feature from the original ones. The parameter *N* denotes the number of original features. Each number in the parentheses denotes the sequence number of the production rule.

For example, consider the chromosome $x=\left[ 9,8,6,4,16,10,17,23,8,14\right]$ and *N* = 3. The steps to produce the expression $f(x)=x_{2}+\cos {\left( x_{3}\right) }$ are listed in Table 1. For the feature construction approach the process to produce $N_{f}$ features from the original set of features is as follows:

1. Every chromosome *Z* is split into $N_{f}$ parts. Each part $g_{i}$ will be used to construct a feature.
2. For every part $g_{i}$ construct a feature $t_{i}$ using the grammar given in Fig. 2
3. Create a mapping function
  1 $$\begin{aligned} G(\overrightarrow{x},Z)=\left( t_{1}\left( \overrightarrow{x},Z\right) ,t_{2}\left( \overrightarrow{x},Z\right) ,\ldots ,t_{N_{f}}\left( \overrightarrow{x},Z\right) \right) \end{aligned}$$
  where $\overrightarrow{x}$ is a pattern from the original set and *Z* is the chromosome.

**Table 1:** Steps to produce a valid expression from the BNF grammar

String	Chromosome	Operation
<expr>	9,8,6,4,16,10,17,23,8,14	$9\mod 3=0$
(<expr$> <$op$> <$expr>)	8,6,4,16,10,17,23,8,14	$8\mod 3=2$
(<terminal$> <$op$> <$expr>)	6,4,16,10,17,23,8,14	$6\mod 2=0$
(<xlist$> <$op$> <$expr>)	4,16,10,17,23,8,14	$4\mod 3=1$
(x2<op$> <$expr>)	16,10,17,23,8,14	$16\mod 4=0$
(x2+<expr>)	10,17,23,8,14	$10\mod 3=1$
(x2+<func>(<expr>))	17,23,8,14	$17\mod 4=1$
(x2+cos(<expr>))	23,8,14	$23\mod 2=1$
(x2+cos(<terminal>))	8,14	$8\mod 2=0$
(x2+cos(<xlist>))	14	$14\mod 3=2$
(x2+cos(x3))		

### Proposed Algorithm

A new artificial set of features is derived from the original feature set using a combination of grammatical evolution and a nonlinear classifier such as a radial basis function (RBF) neural network. The artificial features are constructed from the original ones using a non-linear mapping. A new train and test set is constructed based on the original sets using the newly constructed features.

The main steps of the proposed procedure are outlined below:

1. Initialization step
  - *Set*: iter=0, $N_{f}$ the number of desired features and $N_{c}$ as the number of chromosomes.
  - *Initialize*: chromosomes $X_{i},i=1\ldots N_{c}$. The chromosomes are initialized randomly as vectors of integers in the range $[-255,255]$.
  - *Set*: ITERMAX as the maximum number of allowed generations.
  - *Set*: $p_{s}$ as the selection rate and $p_{m}$ the mutation rate. Both rates are in the range [0, 1]
2. Calculate the fitness $f_{i}$ for every chromosome of the population:
  - *Create*: $N_{f}$ features from the original data, through the grammatical evolution procedure given in SubSect. 3.2.
  - *Train*: a classification model *C* using the MSE error defined by the quantity:
    2 $$\begin{aligned} E=\sum _{j=1}^{M}\left( C_{i}\left( x_{j}\right) -t_{j}\right) ^{2} \end{aligned}$$
    where *M* denotes the number of input patterns, $x_{j}$ is the *j* pattern of created data and $t_{j}$ is the desired output.
  - *Assign* the train error *E* to fitness value $f_{i}$
3. Genetic Operators
  - *Selection procedure*: The chromosomes are sorted in descending order according to their fitness value. The first $\left( 1-p_{s}\right) \times N_{c}$ chromosomes are transferred to the next generation. The rest of the chromosomes are substituted by offsprings created through the crossover procedure using tournament selection for selecting parents and one point crossover to mate the selected parents.
  - *Mutation procedure*: For every element of each chromosome a random number *r* in range $\left[ 0,1\right]$ is produced. If $r\le p_{m}$ then the corresponding element is randomly altered.
4. Set iter=iter+1
5. if $$\text{ iter } \ge \text{ ITERMAX }$$ then
  - *Obtain * the best value in the population, denoted as $f_{l}$ for the corresponding chromosome $x_{l}$
  - Terminate
6. else goto Step 2.

## Experiments

To understand the dynamics of the proposed methodology a series of experiments were performed and comparative studies were made. In the first set of experiments, the proposed methodology was applied to the 7-category data set presented earlier. In the second series of experiments an attempt was made to predict mortality due to the pandemic for a number of selected countries. In the third set of experiments, the proposed technique was applied on data freely available online from the Israeli Ministry of Health.

### Experiments with the Seven Categories dataset

We have conducted experiments using the Seven Categories dataset. In all experiments the size of the population was set to 500, the mutation rate was set to 5% and the selection rate was set to 10%. In all the experiments 10 fold cross validation was used. The original dataset was divided randomly into 10 sets of train and test. In every set the train data was about 90% of the original data and the remaining was the test. This method was used with success during the past years in a variety of scientific fields [18–20]. An important advantage of this technique is that all patterns are used in both the training set and the test set only once. In addition, this technique is quite general and does not require previous knowledge of the dataset but also does not require any special manipulation to create each fold.

All the experiments were conducted 30 times and averages were taken. In the experiments, we have used Radial Basis Function neural networks to construct new features from the original ones. The evaluation of the produced features was made using the FunctionParser programming library [21]. The results are reported in Table 2. The following methods were used during the comparisons:

1. *MLP back propagation*: A neural network with 10 hidden nodes is used. The network is trained using the Back Propagation method [22].
2. *MLP Bfgs*: A neural network with 10 hidden nodes is used. The network is trained using the Broyden Fletcher Goldfarb Shanno (BFGS) local optimization method and more specifically a variant due to Powell [23].
3. *MLP genetic*: A neural network with 10 hidden nodes is used. The network is trained using a genetic algorithm [24] with 500 chromosomes and 500 generations.
4. *KNN*: The simple algorithm of k-nearest neighbors (KNN) [25] was used as test case. In the current work k was set to 3.
5. *SVM*: The method of Support Vector Machines (SVM) [26] was used in this dataset.
6. *PCA*: A Principal Component Analysis (PCA) method obtained from [27] with two constructed features.
7. *MRMR*: Minimum Redundancy Maximum Relevance Feature Selection (MRMR) [28] method for two features.
8. *Feature construction*: (the proposed method) A feature construction method initially outlined in [2] was used using two constructed features. The newly created features are used as input to a neural network with 10 hidden nodes and trained using the Genetic Algorithm used above.

**Fig. 3:**
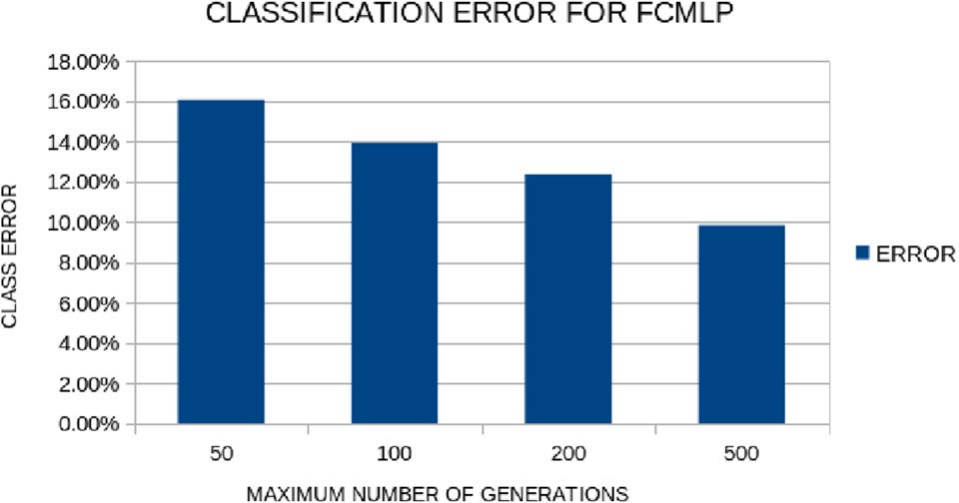
Average classification error for the proposed method for different values for the maximum number of generations allowed

**Fig. 4:**
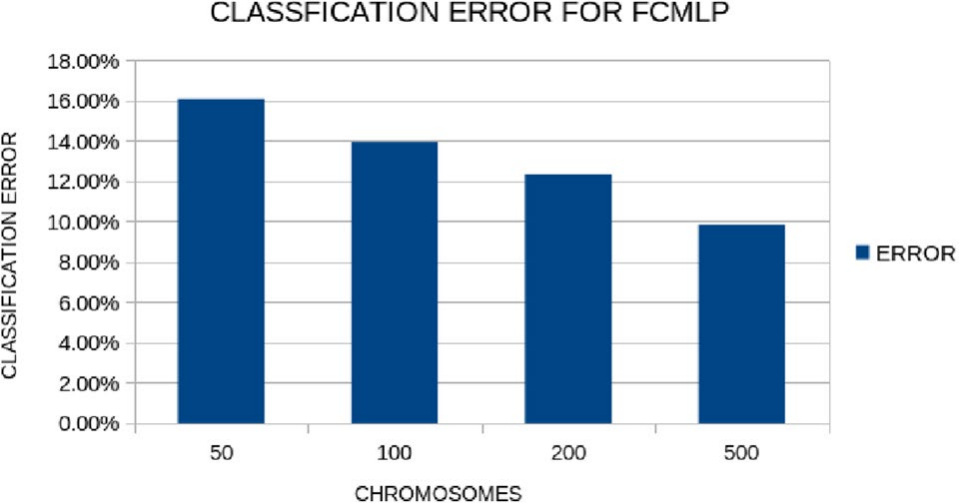
Average classification error for the proposed method for different values for the number of chromosomes in the Grammatical Evolution procedure

Also, two additional experiments were conducted: one with the maximum number of generations allowed and the other with the number of chromosomes that participate in the Grammatical Evolution process. The results for the first experiment our outlined graphically in Fig. 3 and for the second experiment in Fig. 4. In the case of the number of generations, the average error decreases as the maximum number of generations increases, although 200 generations are probably enough to achieve a high learning rate. Similarly, in the case of the number of chromosomes the average error decreases as their number increases.

The proposed method outperforms the other machine learning techniques significantly in terms of classification error, which proved to be below 10instead of seven features used by the other methods.

**Table 2:** Experimental results for the seven categories dataset

Method	TEST ERROR
MLP back propagation	46.62%
MLP Bfgs	23.48%
MLP genetic	21.92%
KNN	20.00%
SVM	42.38%
PCA	37.89%
MRMR	44.90%
Feature construction	9.84%

### Experiments for Mortality Prediction

The results for the case of mortality prediction (second dataset) are shown in Table 3. All the experiments were conducted using 10-fold cross-validation and each experiments was conducted 30 times and averages were taken. The numbers in cells denote average regression error. The columns in this table stand for the following:

- *Country*: the name of the country.
- *MLP*: the results using a neural network with 10 hidden nodes trained with BFGS.
- *RBF*: the results taken using a Radial Basis Function with 10 processing units.
- *FC MLP*: the results from applying an MLP in the dataset produced by constructing one feature from the original ones.
- *FC RBF*: the results from applying an RBF in the dataset produced by constructing one feature from the original ones.

**Fig. 5:**
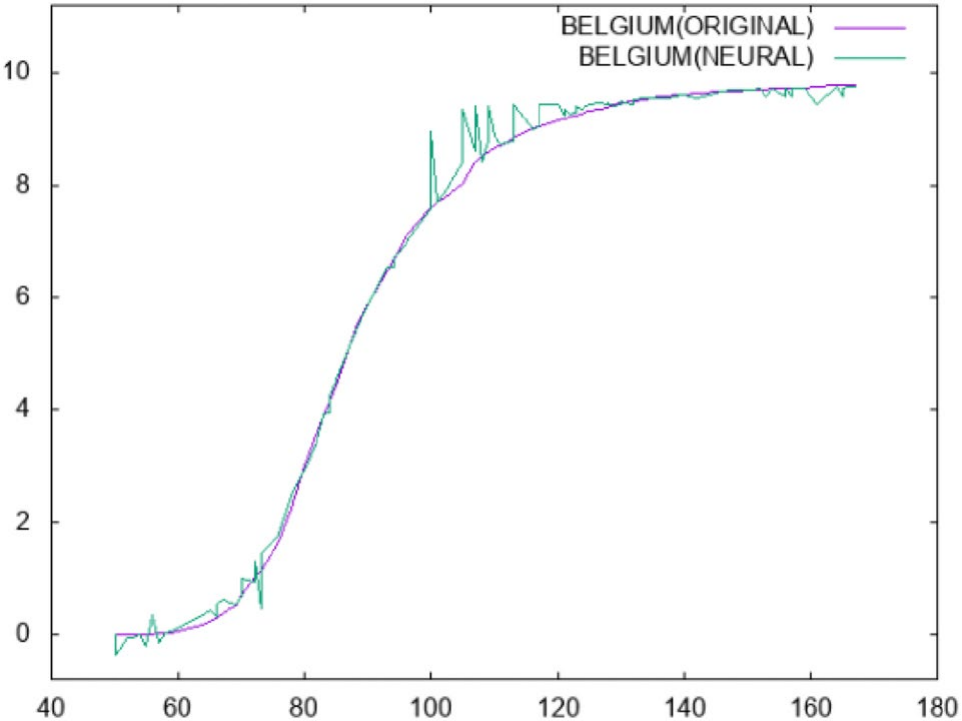
Prediction for mortality in Belgium using a Neural Network with 10 hidden nodes

**Fig. 6:**
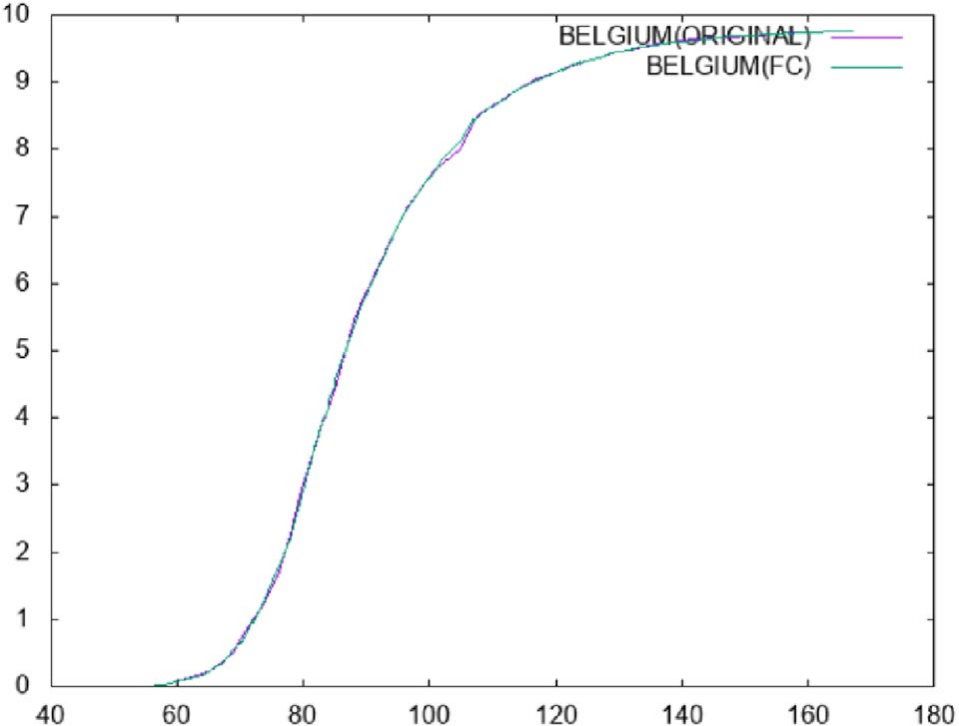
Prediction for mortality in Belgium using the proposed method of Feature Construction

Again, the proposed feature construction method outperforms MLP and RBF in terms of average regression error. Also, in Fig. 5 the plot of the deaths in Belgium for the first 180 days of the pandemic is plotted against the estimation made by a neural network with 10 hidden nodes. The neural network was trained using the BFGS method. In Fig. 6, the deaths in Belgium are estimated using the proposed method. It is evident from the figures that the proposed method managed to estimate the underlying data much more smoothly.

The mortality rate was used as a test case in [8] and in [13] but with different datasets from these discussed in the current paper. In the first work, a LSTM based method is utilized to predict the daily positive cases and the mortality rate for the case of India by using data covering a period of 66 days. The results from this method were very promising; for example they have an error percentage between 1.64% and 8% for predicting the total number of positive cases. In the second method two machine learning approaches (adaptive network-based fuzzy inference system and multi-layered perceptron-imperialist competitive algorithm) were used for data collected from Hungary during March and April 2020. These two machine learning methods were applied to validation set of 9 days and the corresponding RMSE values were 8.32 and 15.25, respectively.

**Table 3:** Experimental results for mortality rate prediction

Country	MLP	RBF	FC MLP	FC RBF
France	0.71	17.73	0.34	1.54
Germany	0.05	0.54	0.04	0.06
Brazil	7.80	84.56	0.60	7.82
Belgium	0.40	0.90	0.02	0.05

### Comparing the Proposed Method with Others

Recently, a machine learning approach [29] was applied to a series of records from the Israeli Ministry of Health. The model was used to predict COVID-19 test results based on binary features such as sex, age. The data are freely available from the relevant https://github.com/nshomron/covidpred and the proposed method was also applied to these data. Again, ten-fold cross-validation was used and the respective results are outlined in Table 4. The symbols in the first column have the same meaning as in Table 3. As in the case of the dataset of seven categories, two distinct features were constructed by the proposed method. Again, the proposed method outperforms the other machine learning techniques in terms of classification error using only two constructed features.

**Table 4:** Experimental results for data from the Israeli Ministry of Health for diagnosis based on symptoms

Method	TEST ERROR
RBF	27.17%
FC MLP	14.67%
FC RBF	15.13%

## Discussion

A method that constructs features from datasets using the Grammatical Evolution technique was proposed here to estimate the mortality rate due to the COVID-19 pandemic. The method was tested on three different cases. In the first case, the method managed to classify the world’s countries in seven mortality classes with a success rate exceeding 90was used to predict the deaths in a series of countries and the results were also very promising. In the third case, a dataset derived from the Israeli Ministry of Health was used to predict the COVID-19 diagnosis.

As can be observed from the results reported, the feature construction method seems to be an auspicious means of predicting the mortality rate due to the COVID-19 disease, and a series of enhancements could be applied to the method such as:

1. Usage of additional machine learning techniques to evaluate the performance of the constructed features such as SVM [30], K-NN [31].
2. incorporation of more advanced stopping rules to terminate the genetic algorithm such as those proposed in [32, 33].
